# Case Report: Identification and functional characterization of a novel heterozygous splice-donor (c.647+1G>A) site mutation in the *SPTB* gene that causes hereditary spherocytosis with hemolytic anemia

**DOI:** 10.3389/fgene.2025.1626155

**Published:** 2025-11-14

**Authors:** Ke Cao, Xiaojuan Luo, Lianlian Liu, Xiaoning Mao, Ruping Liu, Yunsheng Chen, Santasree Banerjee

**Affiliations:** 1 Department of Clinical Laboratory, Shenzhen Children’s Hospital, Shenzhen, Guangdong, China; 2 Department of Genetics, College of Basic Medical Sciences, Jilin University, Changchun, Jilin, China

**Keywords:** hereditary spherocytosis, novel mutation, SPTB gene, hemolytic anemia, splice-donor site mutation

## Abstract

**Objective:**

Hereditary spherocytosis (HS) is an inherited disorder characterized by spherical erythrocytes and abnormalities of several erythrocyte membrane proteins with extreme genotypic and phenotypic heterogeneity. HS patients were clinically diagnosed by the presence of spherical erythrocytes on the peripheral blood smear, hemolytic anemia, jaundice, and splenomegaly, with or without cholelithiasis or gallstones. To date, mutations of five genes (*ANK1, EPB42, SLC4A1*, *SPTA1*, and *SPTB*) have been reported to be associated with different subtypes of HS. Germline mutations of the *SPTB* gene cause autosomal dominant HS (Spherocytosis 2, SPH2), the rarest subtype of HS.

**Methods:**

In this study, we investigated a 10-year-old Chinese girl clinically diagnosed with HS and neonatal hemolytic anemia. The proband’s mother was also identified with HS and hemolytic anemia, but the proband’s father was phenotypically normal. We performed a standard G-banding karyotype to identify structural abnormalities of chromosomes in this proband. Then, we performed whole-exome sequencing and Sanger sequencing to identify the disease-causing variants in this proband. Finally, we functionally characterized the identified novel variant by performing reverse transcription polymerase chain reaction, cDNA sequencing, quantitative real-time polymerase chain reaction (PCR), and Western blot.

**Results:**

Whole exome sequencing identified a heterozygous novel splice-donor-site (c.647 + 1G>A) mutation in the *SPTB* gene in the proband. Sanger sequencing confirmed that the proband inherited this mutation from her mother, while her father was devoid of it. Reverse transcription polymerase chain reaction and cDNA sequencing showed that this novel splice-donor-site (c.647 + 1G>A) mutation causes abolition of the wild-type splice donor site, which leads to the aberrant splicing of SPTB mRNA, followed by the formation of an alternative transcript with complete loss of exon 5. The relative expression of mutated SPTB mRNA was significantly reduced in the proband and her mother compared with her father, showing normal expression of wild-type SPTB mRNA.

**Conclusion:**

Our present study highlighted the significance of whole-exome sequencing as the most promising path to genetic molecular diagnosis for patients with HS.

## 1 Introduction

Hereditary spherocytosis (HS) is an inherited disorder that manifests with spherical erythrocytes predominantly identified on a peripheral blood smear, abnormalities of several erythrocyte membrane proteins, and osmotically fragile spherocytes (Tole et al., 2020). Patients with HS are usually identified with hemolytic anemia, jaundice, and splenomegaly with or without cholelithiasis (Tole et al., 2020). The worldwide incidence of HS varies among different populations (Da Costa et al., 2013). In a Northern European population, the estimated prevalence of HS is 1 in 2000 live births, while in the Chinese population, the incidence of HS is extremely rare, affecting 1 in 100,000 live births (Da Costa et al., 2013; Perrotta et al., 2008). No significant difference in the prevalence of HS was found in male (0.18/1 million) and female (0.19/1 million) neonates (<1 year old) in the Chinese population (Wang et al., 2015). Patients with HS were usually identified with extreme phenotypic heterogeneity, which varies from asymptomatic to a severe form of the disease with skeletal abnormalities, short stature, and delayed puberty (Qin et al., 2020). HS is majorly inherited with an autosomal dominant pattern, while an autosomal recessive mode of inheritance or *de novo* variants have also been reported (Qin et al., 2020; Risinger and Kalfa, 2020). Germline mutations of the *SPTB* gene cause autosomal dominant HS (spherocytosis 2; SPH2; OMIM# 616649), the rarest form among all the HS subtypes (Qin et al., 2020; Delaunay, 2002). However, abnormalities or deficiencies of erythrocyte membrane proteins also showed genotypic and phenotypic heterogeneity among HS patients from populations of different ethnic origins (He et al., 2018; Andolfo et al., 2016).

The *SPTB* gene is located in the long arm of chromosome 14 (14q23.3) with a length of 100 kb. SPTB mRNA comprises 35 exons and is translated to the SPTB (β‐spectrin) protein with 2137 amino acids (Park et al., 2016). β-spectrin comprises five domains, namely, N-terminal calponin homology (CH) 1 and 2 domains (actin binding domain), spectrin domain 1 and 2 (dimerization domain), spectrin domain 3–13 and 16 (spectrin repeat domain), spectrin domain 14 and 15 (ankyrin-binding domain), and spectrin domain 17 (tetramerization domain) (Ipsaro et al., 2009). β‐spectrin is significantly involved in interacting with actin, ankyrin, and band 4.1 protein to organize and stabilize the erythrocyte plasma membrane (He et al., 2018; He et al., 2017; Boguslawska et al., 2017). Hence, a germline mutation in the *SPTB* gene causes the formation of partially or completely nonfunctional β‐spectrin, which may be unable to maintain the biconcave shape of human erythrocytes and causes a deficiency in erythrocyte membrane protein, finally resulting in HS (He et al., 2018; Ma et al., 2018; Fan et al., 2019). Germline mutations in the *SPTB* gene cause an autosomal dominant form of HS (Spherocytosis 2, SPH2), which accounts for approximately 15% of HS patients (Jang et al., 2019). To date, 57 mutations in the *SPTB* gene have been reported to be associated with HS. The majority of *SPTB* mutations are *loss-of-function* mutations, including frameshift, nonsense, or splice-site mutations that often lead to the formation of truncated β-spectrin (Jang et al., 2019).

In our study, we investigated a 10-year-old Han Chinese girl clinically diagnosed with HS and hemolytic anemia. The proband’s mother was also identified with HS and hemolytic anemia, but the proband’s father was phenotypically normal. Whole-exome sequencing identified a heterozygous novel splice-donor-site (c.647 + 1G>A) variant in the *SPTB* gene in the proband. Sanger sequencing confirmed that the proband’s mother also harbored this mutation, while her father did not carry it. Our present study not only expands the mutational spectrum of the *SPTB* gene associated with HS but also highlights the significance of whole-exome sequencing for genetic molecular diagnosis of HS patients.

## 2 Materials and methods

### 2.1 Participants and ethics statement

**FIGURE 1:**
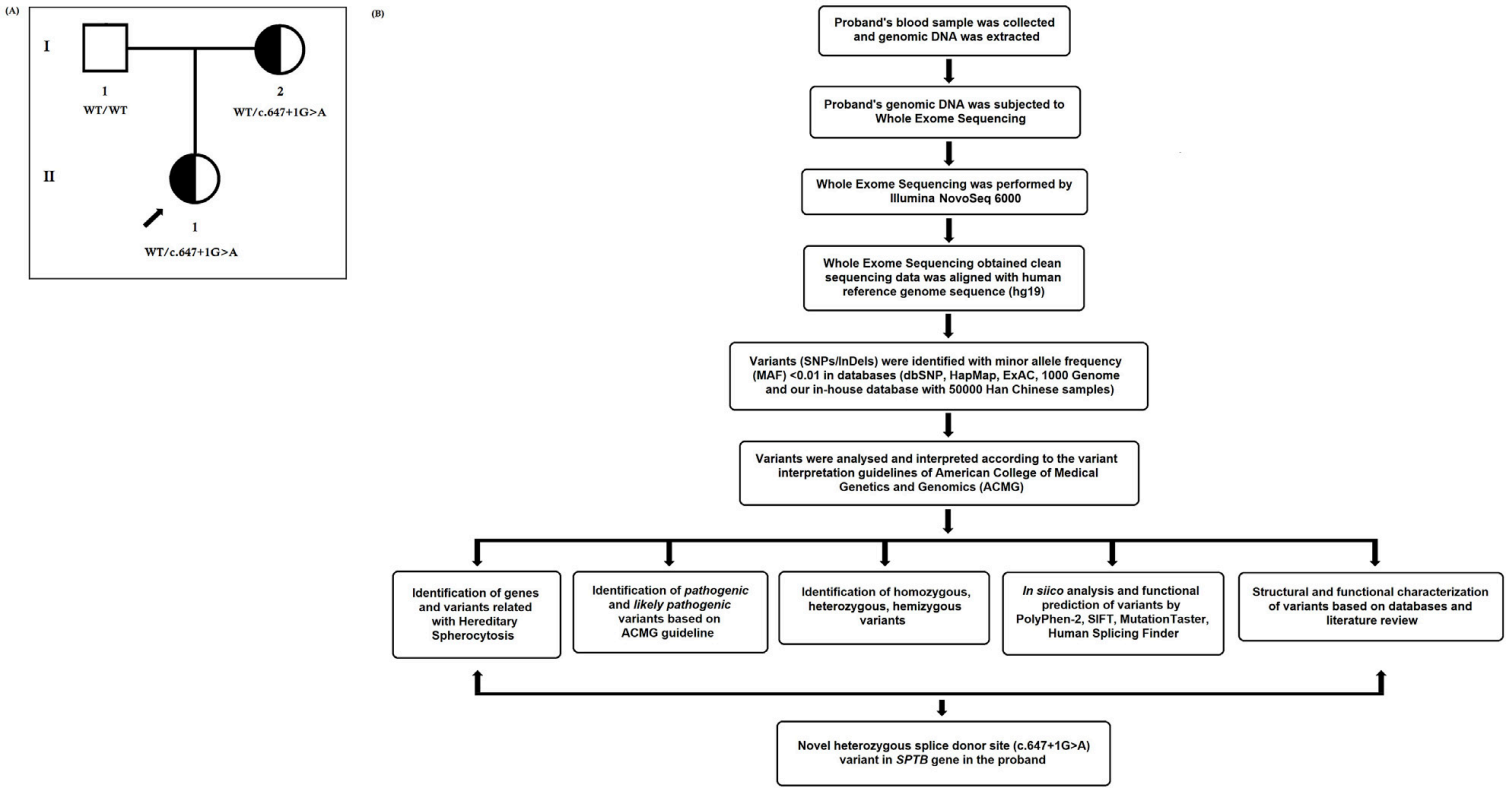
**(A)** Pedigree of the Chinese family with hereditary spherocytosis. Squares and circles denote males and females, respectively. Roman numerals indicate generations. Arrow indicates the proband (II-1). **(B)** Bioinformatics data analysis and interpretation pipeline for whole-exome sequencing.

Here, we investigated a 10-year-old Chinese girl from nonconsanguineous Han Chinese parents (Figure 1A). The proband was clinically diagnosed with HS and hemolytic anemia. Peripheral blood samples were obtained from the proband and her parents at the Department of Clinical Laboratory, Shenzhen Children’s Hospital, No.7019, Yitian Road, Shenzhen, 518,038, Guangdong, China, for clinical diagnosis of the disease. Blood and serum biochemical tests were conducted in the Department of Clinical Laboratory, Shenzhen Children’s Hospital, Shenzhen, China. All participants provided their written informed consent for participating in this study. This study was formally approved by the Ethics Committee of The Shenzhen Children’s Hospital, Shenzhen, China. All procedures were performed following the approved guidelines.

### 2.2 Karyotyping analysis

Standard G-banding karyotype was performed to identify structural abnormalities of chromosomes in this proband (Zhang et al., 2020). We performed G-banding karyotype with the metaphase chromosomes obtained from temporary lymphocyte cultures of the peripheral blood of the proband according to the standard protocol (Zhang et al., 2020).

### 2.3 Chromosome microarray analysis (CMA)

Chromosome microarray analysis (CMA) was performed to identify copy number variations (CNV) in this proband (Zhang et al., 2020). Here, we performed CMA with the Affymetrix CytoScan HD array to identify CNVs in this proband. Affymetrix Chromosome Analysis Suite software (version 3.1) was used for CMA data analysis. Data presentation analysis has been done with reference to the NCBI37/hg19 genome assembly. Finally, the CMA data were analyzed and interpreted by using public databases [UCSC Genome Browser (https://genome.ucsc.edu/), OMIM (https://www.omim.org/), Database of Genomic Variants (DGV, http://dgv.tcag.ca/dgv/), DECIPHER (https://www.deciphergenomics.org/), and ClinGen (https://www.clinicalgenome.org/)] with the copy number threshold of 10 kb and a marker count of ≥50. We used the UCSC Genome Browser (https://genome.ucsc.edu/) to verify the genomic content for all reported calls. OMIM (https://www.omim.org/) has been used to understand the genotype–phenotype genotype–phenotype correlation and inheritance pattern of copy number variations (CNVs). We used DGV (http://dgv.tcag.ca/dgv/) to confirm the human genomic structural variation, such as CNVs, inversions, and deletions. DECIPHER (https://www.deciphergenomics.org/) has been used to compare phenotypic and genotypic data from patients with rare diseases. We used ClinGen to understand the clinical relevance of genes and variants.

### 2.4 Whole exome sequencing

We performed whole exome sequencing for the proband. First, we collected the proband’s peripheral blood and extracted the genomic DNA, according to the manufacturer’s instructions (Zhang et al., 2020; Han et al., 2020). Whole-exome sequencing was performed with the proband’s genomic DNA (Zhang et al., 2020; Han et al., 2020). The sequencing library was prepared by capturing exome sequences using Agilent SureSelect version 6 (Agilent Technologies, Santa Clara, CA, United States). Then, an enriched sequencing library was subjected to whole-exome sequencing by using the Illumina NovaSeq 6000. Next, we obtained sequencing reads, and Burrows–Wheeler Aligner software (http://bio-bwa.sourceforge.net) (version 0.59) was used to align these sequencing reads with GRCh37. p10. Then, the Burrows–Wheeler aligned sequencing reads were again locally realigned by using GATK IndelRealigner (https://gatk. broadinstitute.org). After that, the base quality recalibration was performed with the Burrows–Wheeler aligned sequencing reads by using GATK Base Recalibrator (https://gatk.broadinstitute.org). Next, single-nucleotide variants (SNVs) and insertions or deletions (InDels) were identified by using GATK Unified Genotyper (https://gatk.broadinstitute.org). Then, all these identified variants (both SNVs and InDels) were annotated with the consensus coding sequence database (20,130,630) of the National Center for Biotechnology Information (NCBI) (https://www.ncbi.nlm.nih.gov/CCDS/CcdsBrowse.cgi). Image analysis and base calling were performed through the Illumina pipeline (https://www.illumina.com/informatics/infrastructure-pipeline-setup.html). We designed indexed primers for data fidelity surveillance.

### 2.5 Bioinformatics data analysis and interpretation

All the variants obtained by whole-exome sequencing were collected. Variants were selected based on their minor allele frequency (MAF). We selected variants for bioinformatic data analysis and interpretation if their MAF was < 0.01 in the following public databases: dbSNP (https://www.ncbi.nlm.nih.gov), 1000 Genome Database (http://www.internationalgenome.org), HapMap (https://www.genome.gov), and our in-house database of 50,000 Han Chinese samples (Sherry et al., 2001; Clark et al., 2005; Auton et al., 2015). All these public databases were used to confirm the MAF of identified genetic variations in different populations. According to the MAF (cut off MAF is < 0.01), we can filter out the possible pathogenic variants for specific cases. First, we compared our identified variants with the Human Gene Mutation Database (HGMD, www.hgmd.cf.ac.uk/) (Stenson et al., 2003). HGMD is a comprehensive and updated database containing all the reported variants associated with monogenic disorders. Hence, by comparing our data with HGMD, we can interpret whether the identified genetic variants in our present study are novel variants or were previously reported to be associated with any monogenic diseases. Then, we compared our identified variants with the Online Mendelian Inheritance in Man (OMIM, https://www. omim.org) database (Hamosh et al., 2005). OMIM comprises detailed information on more than 16,000 genes and their associated Mendelian disorders, as well as their pattern of inheritance. Therefore, by comparing our identified genes and their variants with OMIM, we can understand the genotype–phenotype correlation and inheritance pattern of our identified genes and their variants. Next, we compared our identified variants with the Exome Aggregation Consortium (ExAC, http://exac.broadinstitute.org) database (Karczewski et al., 2017). The ExAC database contains whole-exome sequencing data from large-scale sequencing projects from different populations globally. So, by comparing our identified genetic variations with ExAC data, we can confirm whether our identified genetic variations were already reported in some healthy individuals from different populations. After that, we compared our identified genetic variations with the Genome Aggregation Database (gnomAD, https://gnomad.broadinstitute.org) (Karczewski et al., 2020). The gnomAD consists of both whole-genome and whole-exome data from large-scale whole genome or whole exome sequencing projects from different populations worldwide. Therefore, by comparing our identified genetic variations with the gnomAD database, we can understand the frequency of the identified genetic variations in different populations, followed by confirming the possible pathogenicity of our identified genetic variations in this case. Then, we interpreted our identified genetic variations according to the variant interpretation guidelines of the American College of Medical Genetics and Genomics (ACMG) (Richards et al., 2015). Next, we used *in silico* webservers, namely, SIFT (https://sift.bii.a-star.edu.sg/index.html), Polyphen-2 (http://genetics.bwh.harvard.edu/pph2), REVEL (https://genome.ucsc.edu/cgi-bin/hgTrackUi?db=hg19&g=revel), Human Splicing Finder - Version 3.1 (http://www.umd.be/HSF/HSF.html), and MutationTaster (https://www.genecascade.org/MutationTaster2021/#transcript), to predict the possible pathogenic impacts of our identified genetic variations. Finally, after whole-exome sequencing data analysis and interpretation, we selected the most likely genetic variation underlying the disease phenotype in this case.

In addition, “Mutalyzer 2” software was used to confirm the expression of the genetic variant according to the HGVS rules (Lefter et al., 2021). The whole-exome sequencing quality control (QC) data of the proband is described in Table 1. The bioinformatic data analysis and interpretation are schematically presented in Figure 1B.

**TABLE 1:** Quality control (QC) data of whole-exome sequencing of the proband.

Total	Raw reads (All reads)	417698473
	QC fail reads	0
	Raw data (Mb)	21,102.86
	Paired reads	417698473
	Mapped reads	415569832
	Fraction of mapped reads	99.45%
	Mapped data (Mb)	20,984.64
	Fraction of mapped data (Mb)	99.45%
	Properly paired	405484540
	Fraction of properly paired	97.42%
	Read and mate paired	414575354
	Fraction of read and mate paired	99.29%
	Singletons	1148634
	Read and mate map to different chromosome	7802745.00%
	Read1	218232782
	Read2	218232782
	Read1 (rmdup)	129342787
	Read2 (rmdup)	129312329
	Forward strand reads	217281240
	Backward strand reads	217248084
	PCR duplicate reads	186884654
	Fraction of PCR duplicate reads	43.87%
	Map quality cutoff value	20
	Map quality above cutoff reads	381341984
	Fraction of Map Q reads in all reads	91.54%
	Fraction of Map Q reads in mapped reads	91.84%
Target	Target reads	243693624
	Fraction of target reads in all reads	56.93%
	Fraction of target reads in mapped reads	57.19%
	Target data (Mb)	11,846
	Target data Rmdup (Mb)	5983.84
	Fraction of target data in all data	52.42%
	Fraction of target data in mapped data	52.97%
	Len of region	60573987
	Average depth	183.57
	Average depth (rmdup)	98.97
	Coverage (>0×)	99.86%
	Coverage ( ≥ 4×)	99.75%
	Coverage ( ≥ 10×)	99.45%
	Coverage ( ≥ 30×)	96.45%
	Coverage ( ≥ 100×)	72.45%
	Target region count	200,921
	Region covered > 0×	200,368
	Fraction region covered > 0×	99.87%
	Fraction region covered ≥ 4×	99.68%
	Fraction region covered ≥ 10×	99.32%
	Fraction region covered ≥ 30×	96.93%
	Fraction region covered ≥ 100×	72.84%
Flank	Flank size	200
	Len of region (not include target region)	71847296
	Average depth	39.54
	Flank reads	77394593
	Fraction of flank reads in all reads	18.42%
	Fraction of flank reads in mapped reads	18.79%
	Flank data (Mb)	2793.72
	Fraction of flank data in all data	12.72%
	Fraction of flank data in mapped data	12.93%
	Coverage (>0×)	96.93%
	Coverage ( ≥ 4×)	83.87%
	Coverage ( ≥ 10×)	60.00%
	Coverage ( ≥ 30×)	35.87%
	Coverage ( ≥ 100×)	9.93%

### 2.6 Sanger sequencing

We performed Sanger sequencing to validate the disease-causing variant identified by whole-exome sequencing in the proband. Sanger sequencing was performed for the proband and her parents. First, we performed polymerase chain reaction (PCR). Primers were designed according to the reference genomic sequences of the Human Genome from GenBank in NCBI. Sanger sequencing was performed with the PCR products, and data were compared and analyzed.

Whole exome sequencing obtained a heterozygous novel splice-donor site variation, which was validated by Sanger sequencing. Primers used for Sanger sequencing were as follows: F′ 5′- AGC​CTC​TGT​GTG​TGG​TTA​GC -3’; R 5′- CAGCCCTAGCATGAAGCAGA‐3′(Product length: 534 bp; Products on intended targets: >NC_000014.9, *Homo sapiens*, chromosome 14, GRCh38. p14). The reference sequence NM_001024858 of *SPTB* was used.

### 2.7 Reverse transcription polymerase chain reaction (RT-PCR) and cDNA sequencing

Reverse transcription polymerase chain reaction (RT-PCR) and cDNA sequencing were performed to understand the effect of this heterozygous novel splice-donor site mutation of the *SPTB* gene on the splicing event of SPTB mRNA. We isolated and extracted total RNA from the peripheral blood samples of the proband and her parents. Then, we performed RT-PCR and obtained cDNA from the total RNA according to the manufacturer’s protocol (TAKARA RR037A, Shanghai, China). After that, primers were designed to encompass the coding sequence from exon 4 to exon 6. These primers were used to amplify the cDNA from the proband and her parents. After cDNA amplification, the PCR fragments were recovered and subjected to sequencing, followed by data analysis.

### 2.8 Quantitative real-time PCR

Quantitative real-time PCR (q-PCR) has been performed to identify the relative expression of wild type and mutated SPTB mRNA in the proband and her parents. After isolating and extracting the total RNA from the peripheral blood samples of the proband and her parents, we synthesized cDNA from total RNA by performing RT-PCR, according to the manufacturer’s protocol (TAKARA RR037A, Shanghai, China). Then, cDNAs of the proband and her parents were collected for fluorescence quantitative detection by quantitative real-time PCR (q-PCR). The q-PCR was performed with both the target gene and housekeeping gene (GAPDH) for each sample. The relative expression of both wild type and mutated SPTB mRNAs was analyzed by calculating the detected cycle threshold (Ct) value. The sequences of primers for q-PCR were as follows: SPTB- F- 5′- AAG​GTC​GTG​AAA​CAC​GCT​CA -3′, SPTB- R- 5′- TTG​AAT​GCG​TGC​TCC​AGG​TT -3’; GAPDH- F- 5′-TCT​GAC​TTC​AAC​AGC​GAC​ACC-3′, GAPDH- R- 5′-CTG​TTG​CTG​TAG​CCA​AAT​TCG​T-3’.

### 2.9 Western blot

Peripheral blood mononuclear cells (PBMCs) were isolated from whole blood using the density gradient separation method. Cells were lysed by RIPA lysis buffer (#P0013B, Beyotime, China) with added protease and phosphatase inhibitors. The lysate was centrifuged at 13,000 rpm and 4 °C for 10 min, and the cellular protein was collected. Total cellular proteins were separated on an 8% SDS-PAGE analysis and transferred to the Porablot PVDF membrane. Blocking was performed by incubating the membranes with Tris-buffered saline (TBS), pH 7.4, with 0.05% Tween (TBS-T) containing 3% bovine serum albumin (BSA). Membranes were incubated with primary antibodies, rotating at 4 °C overnight, washed three times with TBS-T, and incubated with secondary antibodies for 1 h at room temperature. Primary antibodies [the mouse anti-SPTB (1:1000 in TBS-T; Proteintech, United States of America, #26936-1-AP) and mouse anti-GAPDH (1:1000, Santa Cruz, United States of America, #sc-47724)] and secondary antibody [Goat anti-Mouse IgG HRP (1:2000, Thermos, 31,430)] were used here. Detection of immunoreactive bands was performed using the Thermo Scientific™ SuperSignal™ West Pico PLUS Chemiluminescent Substrate (#34577), according to the manufacturer’s instructions.

## 3 Results

### 3.1 Pedigree and clinical characteristics

**FIGURE 2:**
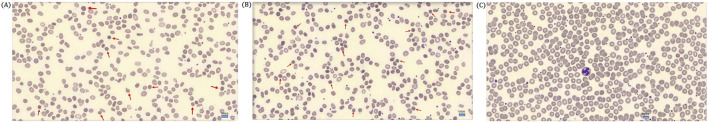
**(A)** Peripheral blood smear of the proband showed spherical erythrocytes. The red arrow marks a spherical erythrocyte (DI-6×0Microscope, ×1000 magnification). **(B)** A peripheral blood smear of the proband’s mother showed few spherical erythrocytes. A red arrow marks a spherical erythrocyte (DI-6×0Microscope, ×1000 magnification). **(C)** A peripheral blood smear of the proband’s father showed no spherical erythrocytes (DI-6×0Microscope, ×1000 magnification).

The proband (II-1) is a 10‐year‐old Han Chinese girl from nonconsanguineous Chinese parents (Figure 1A). She is the first and only child of her parents. The proband clinically manifested HS and hemolytic anemia. Her routine blood test and reticulocyte count results showed increased neutrophil absolute value, neutrophil ratio, red blood cell variation coefficient, red blood cell distribution width, absolute value of reticulocyte, percentage of reticulocytes, reticulum ratio, immature reticulocyte ratio with decreased lymphocyte ratio, eosinophil ratio, red blood cell, hemoglobin, hematocrit, platelet distribution width, and reticulated platelet ratio (Table 2). However, glucose 6-phosphate dehydrogenase activity was found to be normal in the proband (Table 3). The red blood cell osmotic fragility test showed no abnormalities in the proband (Table 3). Hemoglobin A and Hemoglobin A2 were normal, and little elevation of Hemoglobin F was found in the proband (Table 3). Both a direct anti-human globulin test (DAT) and an indirect anti-human globulin test (IAT) were negative for the proband. An antibody screening test for the proband was also negative. The total serum protein test showed an increased level of total bilirubin (both TBIL and indirect bilirubin (IBIL) were elevated) and lactate dehydrogenase, with decreased levels of serum prealbumin, creatinine, serum bicarbonate, and iron in the proband (Table 4). Erythrocyte membrane CD55 and CD59 expressions were completely positive in the proband. In addition, an acidified glycerol dissolution test for 90 s was also positive for the proband. An abdominal ultrasound of the proband revealed diffuse enlargement of the spleen. A peripheral blood smear result showed the presence of spherical erythrocytes (10%) in the proband (Figure 2A).

**TABLE 2:** Routine blood test results of proband, proband’s mother, and proband’s father.

Test item	Proband	Proband’s mother	Proband’s father	References	Unit
Leukocytes	10.5	9.97	5.94	5–12	10^9^/L
Neutrophil absolute value	8.54 **↑**	6.78	3.2	2–7	10^9^/L
Absolute lymphocyte value	1.56	2.63	2.22	0.8–4	10^9^/L
Absolute value of monocytes	0.35	0.42	0.41	0.12–1	10^9^/L
Eosinophil absolute value	0.02	0.04	0.08	0.02–0.5	10^9^/L
Basophil absolute value	0.03	0.1	0.03	0–0.1	10^9^/L
Neutrophil ratio	81.3 **↑**	68	53.9	50–70	%
Lymphocyte ratio	14.9 ↓	26.4	37.4	20–40	%
Mononuclear cell ratio	3.3	4.2	6.9	3–10	%
Eosinophil ratio	0.2 ↓	0.4↓	1.3	0.5–5	%
Ratio of basophils	0.3	1	0.5	0–1	%
Red blood cells	2.99 ↓	3.52	4.45	3.5–5.5	10^12^/L
Hemoglobin	96 ↓	113	152	110–160	g/L
Hematocrit	26.8 ↓	30.9	42.8	30–45	%
Average red blood cell volume	89.6	87.8	96.2	82–99	fL
Average red blood cell hemoglobin content	32.1	32.1	34.2	27–33	pg
Mean red blood cell hemoglobin concentration	358	366↑	355	320–360	g/L
Red blood cell variation coefficient	19.4 ↑	17.4↑	12.1	0–15	%
Red blood cell distribution width	63.1 ↑	54.4↑	42.8	37–50	fL
Platelets	300	212	351	100–300	10^9^/L
Mean platelet volume	8.8	9.2	8.6	7.4–10.4	fL
Platelet packed volume	0.25	0.18	0.28	0.108–0.28	%
Platelet distribution width	8.5 ↓	9.6	8.8	9–17	fL
Large platelet ratio	14.5	19.2	14.2	13–43	%
Absolute value of nucleated red blood cells	0.01	0	0	0–0.5	10^9^/L
Proportion of nucleated red blood cells	0.1	0	0	0–1	/100WBC
Absolute value of reticulocyte	405.4 ↑	287.9↑	70.3	17–70.1	10^9^/L
Percentage of reticulocytes	13.6 ↑	8.2↑	1.6	0.43–1.36	%
Low fluorescence intensity reticulum ratio	85.3↓	83.5↓	93.5	89.9–99.4	%
Medium fluorescence intensity reticulum ratio	11↑	11.4↑	6.3	1.6–9.5	%
High fluorescence intensity reticulum ratio	3.7↑	5.1↑	0.2	0–1.7	%
Immature reticulocyte ratio	14.7↑	16.5↑	6.5	1.6–10.5	%
Reticulum hemoglobin content	34.2	34	35.9	32.1–38.8	pg
Reticulated platelet ratio	0.7↓	1.4	1.1	0.8–6.3	%

**TABLE 3:** Hemoglobin test results of the proband.

Test	Result	References	Unit
Glucose 6-phosphate dehydrogenase activity	1.5	>1	
Red blood cell osmotic fragility	88.5	60–100	%
Hemoglobin H	-	0	%
Hemoglobin Barts	-	0	%
Hemoglobin A	94.9	94.5–96.5	%
Hemoglobin F	2.5↑	0.26–2.3	%
Hemoglobin A2	2.6	2.5–3.5	%
Hemoglobin A2+E	-	0	%
Hemoglobin N	-	0	%
Hemoglobin J	-	0	%
Hemoglobin K	-	0	%
Hemoglobin G	-	0	%
Hemoglobin D	-	0	%
Hemoglobin E	-	0	%
Hemoglobin constant spring	-	0	%

**TABLE 4:** Total serum protein test results of the proband.

Test	Results	References	Unit
Total protein	63.6	46–80	g/L
Albumin	41.2	35–55	g/L
Globulin	22.4	20–30	g/L
Albumin–globulin ratio	1.84	1.1–2.5	
Serum prealbumin	131.7↓	149–307	mg/L
Retinol-binding protein	15.3	5.9–46.6	mg/L
Total bile acid	5	0–15	μmol/L
Total bilirubin	31.4↑	0.9–17.1	μmol/L
Direct bilirubin	11.5↑	0–6.08	μmol/L
Indirect bilirubin	19.9↑	2–17	μmol/L
Alanine transamination	8	0–40	IU/L
Aspartic acid	35	0–40	IU/L
Alkaline phosphatase	115	40–500	IU/L
γ-glutamyl transpeptidase	8	0–50	IU/L
Urea	4.03	2.5–6.0	mmol/L
Creatinine	42.2↓	49–90	μmol/L
Cystatin C	0.87	0.6–1.55	mg/L
Uric acid	275.79	90–420	μmol/L
Creatine kinase	56.8	24–229	IU/L
Creatine kinase isoenzyme	0.3	0–6.8	ng/mL
Lactate dehydrogenase	318↑	140–280	IU/L
Myoglobin	12	<140.2	ng/mL
Troponin	0	<0.018	ng/mL
Sodium	134.9↓	135–146	mmol/L
Potassium	3.96	3.5–5.5	mmol/L
Chlorine	105.5	101–111	mmol/L
Calcium	2.23	2.2–2.7	mmol/L
Serum bicarbonate	17.69↓	20.3–30.3	mmol/L
Magnesium	0.84	0.7–1.15	mmol/L
Iron	6.1↓	9–32.6	μmol/L
Inorganic phosphoric acid	1.45	0.96–2.1	mmol/L
Anion gap	16	6–20	mmol/L
Immunoglobulin G	11.75	5.28–21.9	g/L
Immunoglobulin M	0.83	0.48–2.26	g/L
Immunoglobulin A	1.95	0.51–2.97	g/L
Complement C3	0.68↓	0.7–2.06	g/L
Complement C4	0.23	0.11–0.61	g/L

The proband’s mother (I-2) was a 37-year-old Han Chinese woman who had experienced HS and hemolytic anemia. Her routine blood test and reticulocyte count results showed increased mean red blood cell hemoglobin concentration, red blood cell variation coefficient, red blood cell distribution width, absolute value of reticulocyte, percentage of reticulocytes, and an immature reticulocyte ratio with a decreased eosinophil ratio (Table 2). She was also found to have a decreased level of folic acid (9.64 ng/mL, reference range: >10.6 ng/mL). The total serum protein test showed increased levels of total bilirubin (TBIL and IBIL were elevated), erythropoietin, and soluble transferrin receptor with decreased levels of serum prealbumin and alkaline phosphatase (Table 5). A peripheral blood smear result found few oval erythrocytes and very few spherical erythrocytes (Figure 2B).

**TABLE 5:** Total serum protein analysis result of the proband’s mother.

Test	Result	References	Unit
Total protein	73.8	46–80	g/L
Albumin	43.9	35–55	g/L
Globulin	29.9	20–30	g/L
Albumin/globulin ratio	1.47	1.1–2.5	
Serum prealbumin	196.7↓	200–400	mg/L
Retinol-binding protein	33.9	25–70	mg/L
Total bile acid	3	0–15	μmol/L
Glycine	0.68	0–6.7	mg/L
Total bilirubin	36.9↑	5–21	μmol/L
Direct bilirubin	9.9↑	0–6.06	μmol/L
Indirect bilirubin	27↑	2–17	μmol/L
Alanine aminotransferase	4	0–40	IU/L
Aspartate aminotransferase	11	0–40	IU/L
Alkaline phosphatase	34↓	40–500	IU/L
γ-glutamyl transpeptidase	8	0–50	IU/L
Iron	15.4	9–32.6	μmol/L
Ferritin	29.36	10–291	ng/mL
Serum transferrin (immunoturbidimetric method)	2.05	2–3.6	g/L
Erythropoietin	40.9↑	1.46–31.88	mIU/L
Soluble transferrin receptor	4.3↑	0.76–1.76	mg/L

The proband’s father (I-1) was a 40-year-old Han Chinese man. He is phenotypically normal. A routine blood test result of the proband’s father showed no abnormalities (Table 2). A peripheral blood smear result found no spherical erythrocytes (Figure 2C).

According to biochemical tests and the peripheral blood smear experiment, the proband was clinically diagnosed with HS with hemolytic anemia according to the diagnostic thresholds for HS in practice guidelines.

### 3.2 Karyotype and chromosomal microarray analyses

**FIGURE 3:**
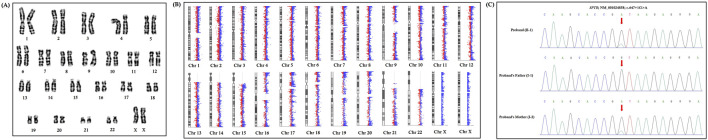
Karyotype analysis, chromosome microarray analysis, and Sanger sequencing. **(A)** Karyotype analysis identified no chromosomal structural abnormalities in the proband (46, XX). **(B)** Chromosome microarray analysis identified no pathogenic copy number variations (CNVs) in the proband. **(C)** Partial DNA sequences in the *SPTB* gene were obtained by Sanger sequencing of the family. The reference sequence NM_001024858 of the *SPTB* gene was used.

We identified no chromosomal structural abnormalities in the proband by karyotype analysis (46, XX) (Figure 3A). We have not found any pathogenic copy number variations (CNVs) in the chromosomes of the proband by CMA (Figure 3B).

### 3.3 Identification of a splice-donor site mutation in the *SPTB* gene

Whole-exome sequencing identified a novel heterozygous splice-donor site mutation (c.647 + 1G>A) in the first base of intron 5 of the *SPTB* gene in this proband (Figure 3C). Sanger sequencing also confirmed that this heterozygous novel mutation was also present in the proband’s mother, while the proband’s father did not harbor this mutation (Figure 3C). Therefore, the proband inherited this heterozygous novel mutation from her mother.

This heterozygous novel mutation (c.647 + 1G>A) has not been found in 100 ethnically matched normal healthy controls. This mutation was also not found in the Human Gene Mutation database (HGMD, www.hgmd.cf.ac.uk/), Online Mendelian Inheritance in Man (MIM, (https://www.omim.org), Exome Aggregation Consortium (ExAC, http://exac.broadinstitute.org), dbSNP (https://www.ncbi.nlm.nih.gov), Genome Aggregation Database (gnomAD, https://gnomad.broadinstitute.org), International Genome Sample Resource (1000 Genome Database, http://www.internationalgenome.org), or our in-house database which consists of ∼50,000 Chinese Han samples.

According to the variant interpretation guidelines of the American College of Medical Genetics and Genomics (ACMG), this novel heterozygous splice-donor site mutation (c.647 + 1G>A) was classified as [PVS1, PS3, PM2, PP1] *“likely pathogenic”* variants (Richards et al., 2015).

In addition, this novel heterozygous splice-donor site mutation (c.647 + 1G>A) was predicted to be a “Broken Wild Type (WT) Donor Site: Alternation of the Wild Type (WT) Donor site,” most probably affecting splicing, by Human Splicing Finder Pro (https://www.genomnis.com/hsf).

This novel heterozygous splice-donor site mutation (c.647 + 1G>A) in the first base of intron 5 of the *SPTB* gene was co-segregated well with the disease phenotype in this family with an autosomal dominant mode of inheritance.

### 3.4 Functional characterization of the splice-donor site mutation

**FIGURE 4:**
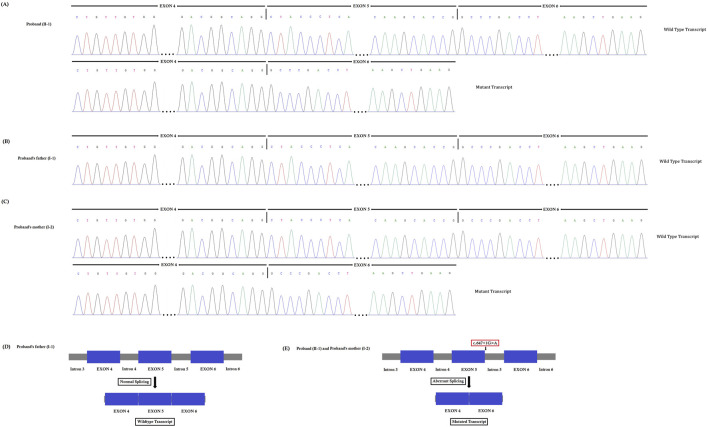
**(A–C)**. Reverse transcription polymerase chain reaction (RT-PCR) and cDNA sequencing. This novel heterozygous splice-donor site (c.647 + 1G>A) mutation in the first base of intron 5 of the *SPTB* gene disrupts the wild type SPTB exon 5 splice donor site. RT-PCR and Sanger sequencing of SPTB cDNA showed normal splicing of exon 4 to exon 6 in the proband’s father, **(B)** while a complete loss of exon 5 (81 bp) was identified in the proband **(A)** and her mother **(C)**. **(D–E)**. Schematic presentation of normal and aberrant splicing of SPTB mRNA.

The novel heterozygous splice-donor site (c.647 + 1G>A) mutation in the first base of intron 5 of the *SPTB* gene disrupts the wild type *SPTB* exon 5 splice donor site. RT-PCR and cDNA sequencing showed normal splicing of exon 4 to exon 6 in the proband’s father, while a complete loss of exon 5 (81 bp) was identified in the proband and her mother (Figures 4A–C). The normal and aberrant splicing of SPTB mRNA upon this splice-donor-site mutation is schematically presented in Figures 4D,E.

### 3.5 Relative expression of SPTB mRNA by quantitative real-time PCR

**FIGURE 5:**
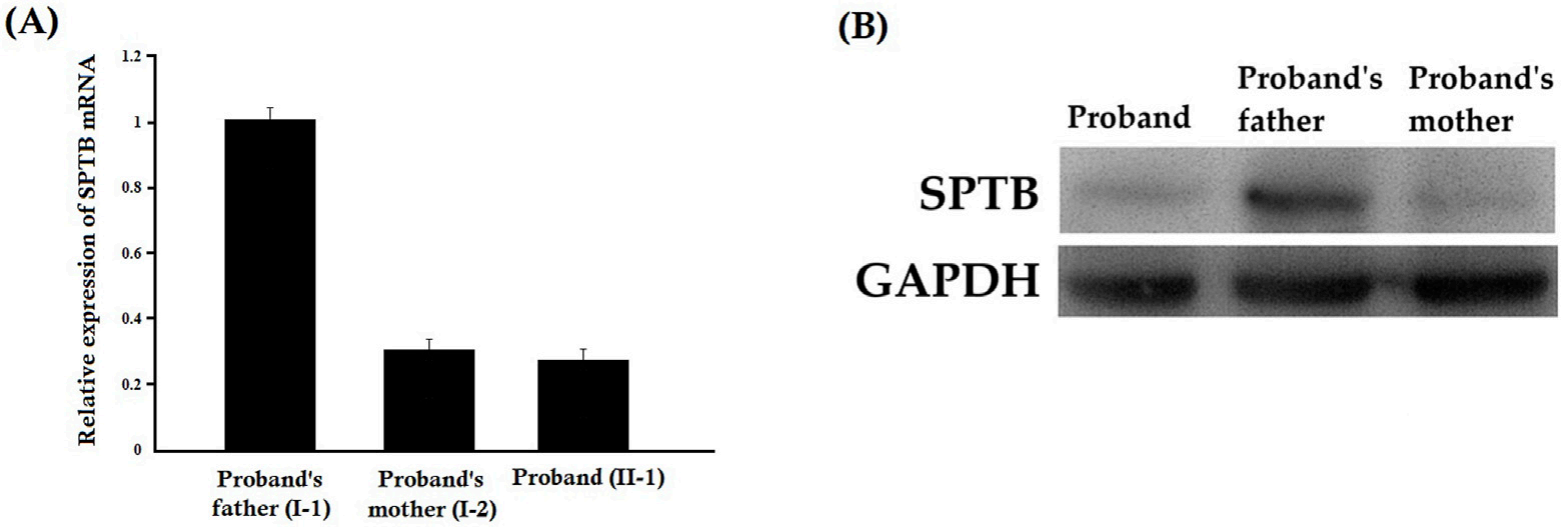
**(A)** Relative expression of SPTB mRNA by quantitative real-time PCR (q-PCR). Relative expression of SPTB mRNA revealed a significantly decreased SPTB transcript level in the proband and her mother, while the proband’s father showed normal expression of SPTB mRNA. Data analysis was performed by using the comparative threshold cycle (2-△△CT) method. **(B)** Relative expression of SPTB protein by Western blot. The relative expression of the SPTB protein showed a significantly decreased mutated SPTB protein level in the proband and her mother, while the proband’s father showed normal expression of wild type SPTB protein.

Relative expression of SPTB mRNA revealed a significantly decreased mutated SPTB transcript level in the proband and her mother, while the proband’s father showed normal expression of wild-type SPTB mRNA (Figure 5A). Our result also suggested that the mutant SPTB transcript was present at a detectable level in the proband and her mother and was not probably degraded by the nonsense-mediated mRNA decay pathway.

### 3.6 Relative expression of the SPTB protein by Western blot

The relative expression of the SPTB protein showed a significantly decreased mutated SPTB protein level in the proband and her mother, while the proband’s father showed normal expression of wild-type SPTB protein (Figure 5B). Notably, a truncated protein product corresponding to the predicted size of the mutant protein (lacking 27 amino acids) was not detected. This suggests that the mutant protein is likely unstable and degraded, and that the clinical phenotype results from haploinsufficiency of the wild-type SPTB allele (Supplementary Figure S1).

## 4 Discussion

Here, we investigated a 10-year-old Chinese girl with hereditary spherocytosis and hemolytic anemia. Whole-exome sequencing and Sanger sequencing identified a novel heterozygous splice-donor site mutation (c.647 + 1G>A) in intron 5 of the *SPTB* gene in this proband. The proband inherited this variant from her mother, while the proband’s father did not carry it.

**FIGURE 6:**
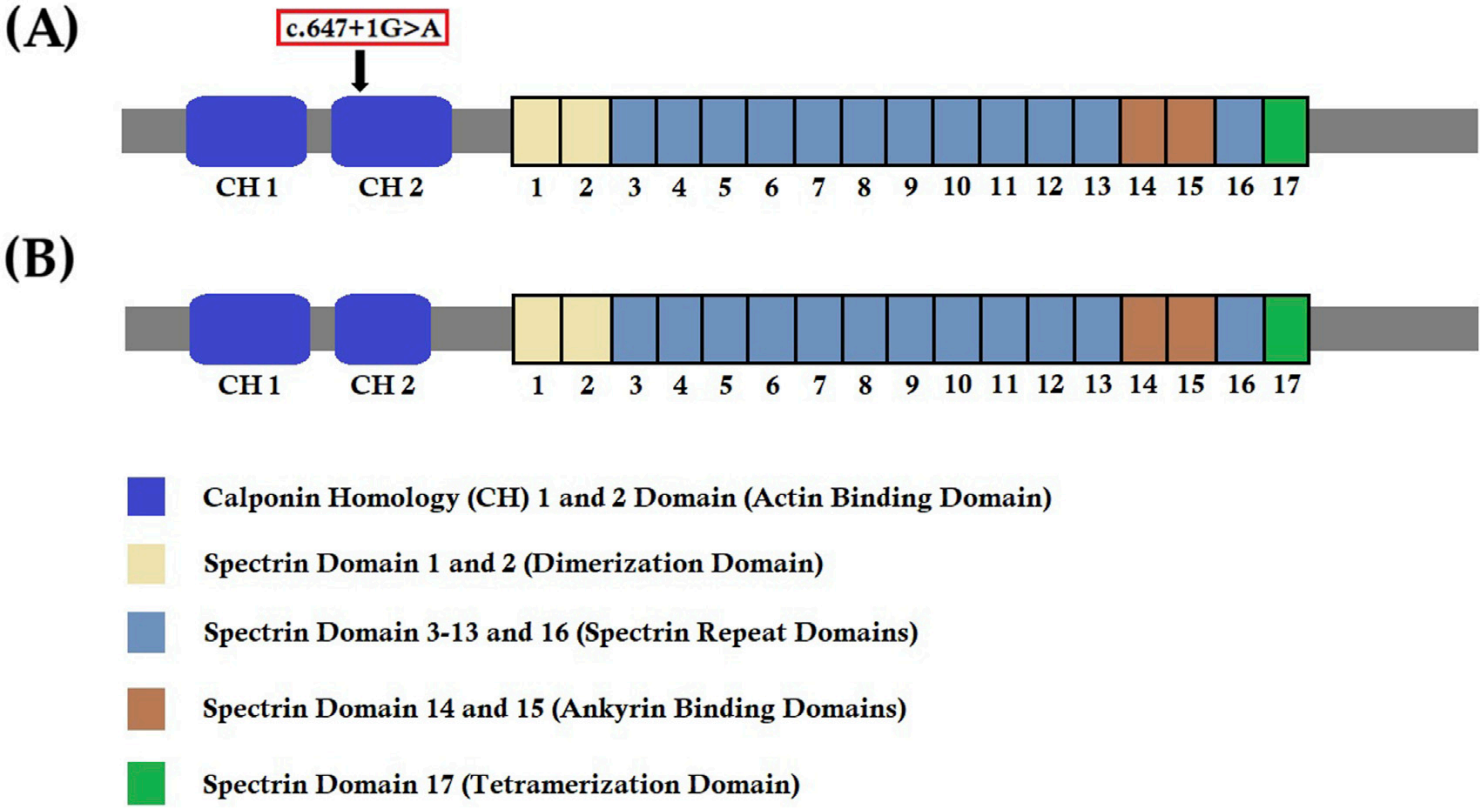
Wild-type and mutant SPTB protein structures. **(A)** Wild-type structure of the SPTB protein showing the location of the identified mutation (c.647G>A) in our study. **(B)** Mutated structure of the SPTB protein after complete loss of exon 5 (81 bp).

We know that any splice-site mutation exerts its effect by causing aberrant splicing of mutated mRNA, followed by the formation of an alternative transcript with partial or complete retention of intron or skipping of exons. In order to understand the effect of this heterozygous novel splice-donor site mutation (c.647 + 1G>A), we performed RT-PCR and subsequent cDNA sequencing of both wild type and mutated SPTB mRNA from the proband and her parents. Our results showed that this novel spice site mutation causes aberrant splicing of SPTB mRNA, followed by the formation of an alternative transcript with complete loss of exon 5. The exon 5 of SPTB mRNA consists of 81 bp, translating into 27 amino acids, constituting part of calponin homology (CH) 2 domains significantly involved in actin binding. Hence, this mutation causes partial loss of the CH2 domain and may reduce the actin-binding ability of the mutated β-spectrin protein. The wild-type β-spectrin protein and the mutated β-spectrin protein are schematically presented in Figures 6 A,B.

Our results also showed the presence of both the wild-type and mutated SPTB transcripts in the proband and her mother, whereas only the wild-type SPTB transcript was found in the proband’s father, suggesting that mutant mRNA was present in detectable levels and was probably not degraded by the nonsense-mediated mRNA decay pathway. In addition, we also found that the relative expressions of mutated SPTB mRNA of the proband and her mother were significantly reduced compared with the wild-type SPTB mRNA of her father. Therefore, this splice-site mutation identified in our study exerts its dominant negative effect as well as causes β-spectrin haploinsufficiency underlying the disease phenotype in this family.

The clinical diagnosis of HS patients is usually performed based on the presence of spherical erythrocytes on the peripheral blood smear, extravascular hemolysis, and physical findings with a positive family history. However, the clinical diagnosis of HS patients is becoming a great challenge due to phenotypic heterogeneity, complex pathophysiology, lack of familial history, and co-occurrence of HS together with other types of hereditary anemias (Fan et al., 2019; Wu et al., 2021; Yang et al., 2019; Aggarwal et al., 2020; Zou et al., 2020). In addition, the prevalence and molecular profile of HS are significantly different in different populations worldwide (Xue et al., 2019; Bogusławska et al., 2021). However, in the Chinese population, a higher rate of misdiagnosis has been reported among HS patients due to mild or moderate clinical manifestations, the presence of other diseases (β-thalassemia or Gilbert syndrome) along with HS, and inconsistent HS genotype and phenotype (He et al., 2018; Andolfo et al., 2016; Roy et al., 2016). Therefore, molecular genetic diagnostics through the application of genomic sequencing technologies will provide proper clinical diagnosis, disease management, and genetic consultation to reduce the risk of disease occurrence in the next generation (Qin et al., 2020; He et al., 2017; Del Orbe Barreto et al., 2016; Schafer et al., 2009; Koboldt et al., 2013; Russo et al., 2018; Xu et al., 2022).

Previous studies showed that the identification of disease-causing mutations in HS patients is usually done by targeted gene panel-based next-generation sequencing or whole-exome sequencing (Tole et al., 2020; Qin et al., 2020; Wu et al., 2021; Yang et al., 2019; Aggarwal et al., 2020; Bogusławska et al., 2021; Xu et al., 2022). However, to date, only two studies have been performed with the application of whole genome sequencing (Nieminen et al., 2021; Du et al., 2021). Whole-exome sequencing provides more sequencing coverage and depth as well as a cost-effective way of identifying disease-causing variants in HS patients more efficiently than targeted gene panel-based next-generation sequencing and whole-exome sequencing. Hence, presently, whole exome sequencing is widely used to identify candidate genes and disease-causing variants in HS patients. Additionally, identification of the mutation will also help us classify the HS and further enable us to provide the clinical or disease management for the patients as well as genetic counseling of the family (Fan et al., 2019; Bolton-Maggs et al., 2012). Genetic counseling and prenatal diagnosis could play a significant role in prenatal and postnatal care of HS patients inheriting diseases causing germline mutation in the *SPTB* gene (Ribeiro et al., 2000). In this study, we also performed whole exome sequencing to identify the mutational characteristics of a Chinese family with HS and rapidly discovered the causative genotype in this family, thus providing genetic information for reproductive risk consultation.

HS patients with germline mutations in the *SPTB* gene usually present with extreme phenotypic heterogeneity. We recommend treatment or therapeutic interventions for HS patients according to their clinical symptoms. HS patients clinically manifest with an enlarged spleen, jaundice, gallstones, and hemolytic anemia. We recommend phototherapy or light treatment for a newborn with jaundice and recommend blood transfusion for treating anemia. Splenectomy or surgical removal of the spleen is also recommended for HS patients. In addition, surgical removal of the gallbladder, or cholecystectomy, has been recommended for HS patients. We also recommend iron chelation therapy for removing excess iron in HS patients with iron overload due to regular blood transfusions. These are the recommended disease management practices for HS patients. Here, we recommend a blood transfusion for the proband if her hemoglobin is lower than 60 g/L. We usually performed blood transfusions for the proband and her mother 1–2 times a year.

β-spectrin protein interacts with the α-spectrin protein to form a heterotetramer (α2β2), which finally results in the formation of a dense erythrocyte-membrane-skeleton network and connects it to the lipid bilayer for maintaining the normal structure of the erythrocyte (Maddala et al., 2016; Bennett and Lorenzo, 2016; Meng et al., 2019). β-spectrin is a rate-limiting protein and plays a key role in the formation of the α2β2-heterotetrameric network. The C-terminal region of β-spectrin attaches to the α2β2-heterotetrameric network with the lipid bilayer (Meng et al., 2019). β-spectrin also binds with actin, ankyrin, and band 4.1 protein and is significantly involved in the formation of the cytoskeletal superstructure of the erythrocyte cell membrane, as well as stabilizing it (Maddala et al., 2016; Bennett and Lorenzo, 2016). β-spectrin binds with ankyrin to anchor the cytoplasmic face of the cell membrane (Bennett and Lorenzo, 2016; Ipsaro and Mondragon, 2010). Hence, β-spectrin helps to maintain the deformability of erythrocytes in capillaries through membrane skeleton cohesion (Meng et al., 2019). β-spectrin also maintains the biconcave shape of human erythrocytes, regulates the plasma membrane components, and maintains the lipid asymmetry of the plasma membrane (Pishesha et al., 2014; Narla and Mohandas, 2017; Machnicka et al., 2014). Hence, germline mutation in the *SPTB* gene results in a decrease in both the cohesion of the membrane skeleton and the surface area of the membrane, leading to the formation of spherical erythrocyte, which finally causes decreased deformability of erythrocytes and premature destruction of erythrocytes in the spleen (He et al., 2018; Meng et al., 2019; Shin et al., 2018; Christensen et al., 2014).

Here, we investigated all the previous reports and found that the *SPTB* gene mutation-associated HS accounts for 20%–25% of all HS cases in Europe and the United States, while it showed a potentially higher rate in China (Perrotta et al., 2008; Wang et al., 2018; Hassoun et al., 1997). In China, germline heterozygous mutations in the *SPTB* gene have been identified in 45% of HS patients (Qin et al., 2020; Wang et al., 2018; Wang et al., 2020). These studies suggested that HS patients showed different geographical distribution of mutations in the *SPTB* gene and, most interestingly, *SPTB* is the major gene among HS patients in the Chinese population. We also investigated the *SPTB* gene mutation databases (Human Gene Mutation Database, http://www.hgmd.cf.ac.uk; Leiden Open Variation Database, LOVD v.3.0, https://www.lovd.nl/) and found that HS-associated *SPTB* mutations include nonsense, frameshift, and splice-site mutations, most often exerting a dominant negative effect on the splicing event of SPTB mRNA, which finally results in the formation of truncated β-spectrin (Maciag et al., 2009; Li et al., 2025). However, we found no common *SPTB* mutations or mutation hotspots in the *SPTB* gene. So far, all the identified *SPTB* mutations associated with HS are unique and occur in individual families. Hence, we could not predict the severity of the disease based on the type or location of the *SPTB* mutations (Tole et al., 2020; Agarwal et al., 2023; Wang et al., 2024; Qin et al., 2025). However, to date, genotype–phenotype association studies have been rarely reported in HS patients and require further investigation.

## 5 Conclusion

In conclusion, in the present study, we investigated a Chinese girl with hereditary spherocytosis and hemolytic anemia. Whole-exome sequencing identified a heterozygous novel splice-site mutation (c.647 + 1G>A) in the *SPTB* gene in the proband. Sanger sequencing confirmed that the proband inherited this heterozygous novel splice-donor site mutation from her mother, while her father did not carry this mutation. Our study confirmed the functional impact of this identified mutation at the molecular level and expanded the spectrum of *SPTB* mutations associated with HS. Our results also significantly highlighted the application of whole-exome sequencing for clinical diagnosis of patients with HS, which may contribute to the clinical management and genetic counseling of HS patients.

Databases and Web servers used:

1000 Genome Database: http://www.internationalgenome.org
Burrows–Wheeler Aligner software: http://bio-bwa.sourceforge.net, version 0.59
ClinGen: https://www.clinicalgenome.org/
dbSNP: https://www.ncbi.nlm.nih.gov
Database of Genomic Variants (DGV): http://dgv.tcag.ca/dgv/
DECIPHER: https://www.deciphergenomics.org/
Exome Aggregation Consortium (ExAC): http://exac.broadinstitute.org
GATK Base Recalibrator: https://gatk.broadinstitute.org
GATK IndelRealigner: https://gatk.broadinstitute.org
GATK Unified Genotyper: https://gatk.broadinstitute.org
Genome Aggregation Database (gnomAD): https://gnomad.broadinstitute.org
HapMap: https://www.genome.gov
Human Gene Mutation Database (HGMD): www.hgmd.cf
Human Splicing Finder - Version 3.1: http://www.umd.be/HSF/HSF.html
Human Splicing Finder Pro: https://www.genomnis.com/hsf
Illumina pipeline: https://www.illumina.com/informatics/infrastructure-pipeline-setup.html
Leiden Open Variation Database, LOVD v.3.0: https://www.lovd.nl/
MutationTaster: https://www.genecascade.org/MutationTaster2021/#transcript
National Center for Biotechnology Information (NCBI): https://www.ncbi.nlm.nih.gov/CCDS/CcdsBrowse.cgi
Online Mendelian Inheritance in Man (OMIM): https://www.omim.org
Polyphen-2: http://genetics.bwh.harvard.edu/pph2
REVEL: https://genome.ucsc.edu/cgi-bin/hgTrackUi?db=hg19&g=revel
SIFT: https://sift.bii.a-star.edu.sg/index.html
UCSC Genome Browser: https://genome.ucsc.edu/)

## Data Availability

The datasets presented in this study can be found in online repositories. The names of the repository/repositories and accession number(s) can be found below: https://bigd.big.ac.cn/gsa-human/browse/HRA010244,HRA010244.
