# Supplementary figures and images for: Case Report: Identification and functional characterization of a novel heterozygous splice-donor (c.647+1G>A) site mutation in the SPTB gene that causes hereditary spherocytosis with hemolytic anemia
Source: Front Genet. 2025 Nov 14;16:1626155. doi: 10.3389/fgene.2025.1626155 (PMC12659992; doi:10.3389/fgene.2025.1626155)

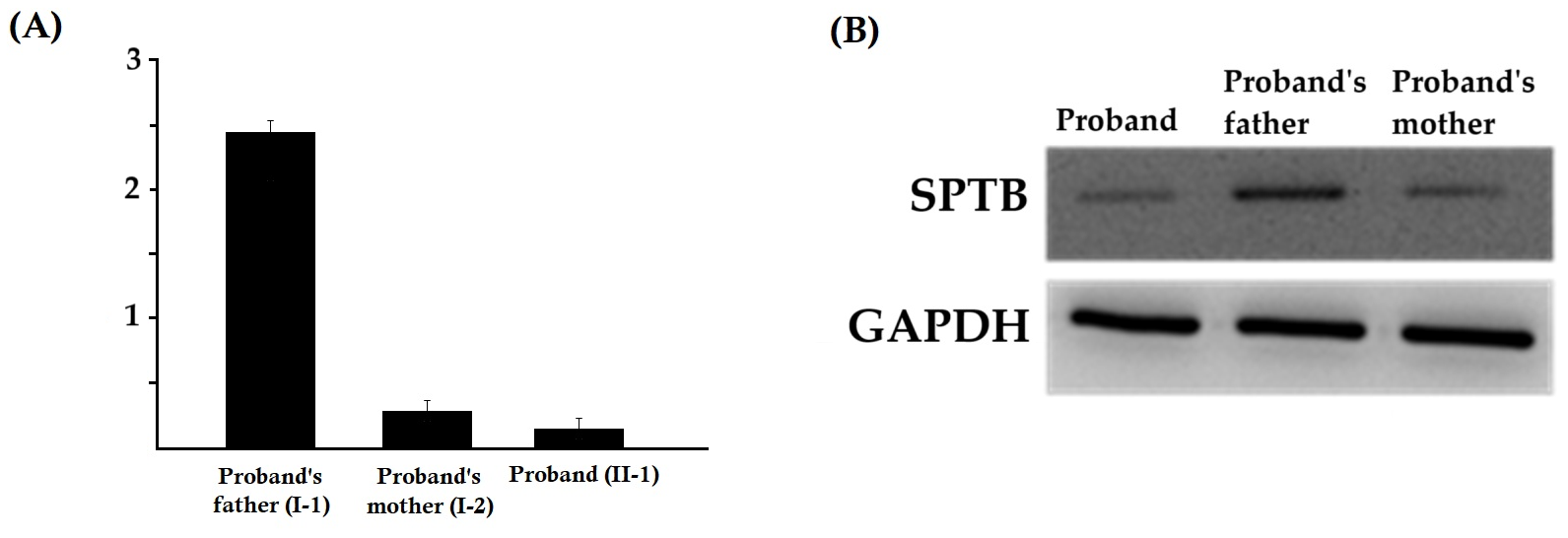

Supplement: Supplementary file 1 [file Image1.tif]
